# Enhancement of Neocortical-Medial Temporal EEG Correlations during Non-REM Sleep

**DOI:** 10.1155/2008/563028

**Published:** 2008-06-15

**Authors:** Nikolai Axmacher, Christoph Helmstaedter, Christian E. Elger, Juergen Fell

**Affiliations:** ^1^Department of Epileptology, University of Bonn, 53105 Bonn, Germany; ^2^Life and Brain Academic Research, University of Bonn, 53105 Bonn, Germany

## Abstract

Interregional interactions of oscillatory activity are crucial for the integrated processing of multiple brain regions. However, while the EEG in virtually all brain structures passes through substantial modifications during sleep, it is still an open question whether interactions between neocortical and medial temporal EEG oscillations also depend on the state of alertness. Several previous studies in animals and humans suggest that hippocampal-neocortical interactions crucially depend on the state of alertness (i.e., waking state or sleep). Here, we analyzed scalp and intracranial EEG recordings during sleep and waking state in epilepsy patients undergoing presurgical evaluation. We found that the amplitudes of oscillations within the medial temporal lobe and the neocortex were more closely correlated during sleep, in particular during non-REM sleep, than during waking state. Possibly, the encoding of novel sensory inputs, which mainly occurs during waking state, requires that medial temporal dynamics are rather independent from neocortical dynamics, while the consolidation of memories during sleep may demand closer interactions between MTL and neocortex.

## 1. INTRODUCTION

Memory consolidation has been suggested to occur in two subsequent steps: while initial encoding depends on the integrity of the medial temporal lobe (MTL) (e.g., [1, 2]) and is linked to the formation of transient assemblies via fast synaptic plasticity in the entorhinal cortex and hippocampus, subsequent consolidation requires the transfer of information to the neocortex, where more permanent networks are built [3–6]. During both waking state and sleep, communication of the hippocampus with the neocortex mainly proceeds via polymodal regions within the rhinal cortex [7]. The rhinal cortex receives rich input from modality-specific regions in higher-order sensory areas which are located in the inferior temporal neocortex. The inferior temporal cortex is the final processing stage of the ventral visual stream and comprises object-specific regions such as the fusiform face area [8, 9]. Recently, Klopp and colleagues [10] used intracranial EEG to show that activity within this area was coherent with activity in widespread brain regions selectively during face processing. Furthermore, fusiform and rhinal cortices are synchronized during memory retrieval [11]. These data indicate that during waking state, interactions between sensory and medial temporal regions are required.

In an influential model, Buzsáki [3] hypothesized that during waking state, and particularly during exploratory phases, information is transferred into the hippocampus and induces rapid though transient forms of synaptic plasticity (see also [12]). Physiologically, strong cholinergic inputs during waking state inhibit feedback excitation in the CA3 region of the hippocampus and induce *θ* (4–8 Hz) and *γ* (20–44 Hz) oscillations; during sleep, a reduced level of acetylcholine leads to disinhibition of hippocampal pyramidal cells, which consequently engage in highly synchronized population bursts [4]. These bursts have been linked to replay of previously acquired information and transfer into the neocortex, where more stable representations are being built [5]. Taken together, these studies suggest a bidirectional information flow between MTL and neocortex, with transmission from the neocortex into the hippocampus during exploration and from the hippocampus into the neocortex during rest and sleep.

The close connection between memory consolidation and sleep [13] suggests that interactions of neocortical and medial temporal EEG activity also undergo circadian fluctuations. However, there are very few data on the interaction between neocortical and medial temporal EEG oscillations during waking state and sleep in humans, partly due to the difficulty of obtaining EEG recordings from the human MTL. Neocortical as well as MTL *θ* and *γ* oscillations were suggested to underlie declarative memory encoding and retrieval (e.g., [12, 14–16]. On the other hand, neocortical slow wave activity (i.e., <4 Hz activity, which includes both *δ* activity between 1 and 4 Hz and slow oscillations <1 Hz) as well as sleep spindles in the *β* (12–20 Hz) range, was shown to be important for the consolidation of previously acquired declarative memories during sleep (e.g., [17, 18]). It is unknown, however, whether there are state specific correlations between the amplitudes of neocortical and medial temporal EEG oscillations. To investigate this question, we analyzed scalp and intracranial EEG recordings in patients with pharmacoresistant focal epilepsy undergoing presurgical evaluation for exact localization of the seizure onset zone.

## 2. MATERIALS AND METHODS

During presurgical evaluation, polysomnography and intracranial EEG were recorded from ten patients (six women; mean age 40.1 ± 22.6 years) with pharmacoresistant unilateral temporal lobe epilepsy. Mean duration of epilepsy was 21.4 ± 11.3 years. Scalp EEG was recorded from positions Cz, C3, C4, and O1 (10–20 system). Electro-ocular activity was registered at the outer canthi of both eyes, and submental electromyographic activity was acquired with electrodes attached to the skin. Scalp as well as depth electroencephalograms were referenced to linked mastoids, bandpass filtered (0.01 Hz (6 dB/octave) to 70 Hz (12 dB/octave)), and recorded with a sampling rate of 200 Hz.

Multicontact depth electrodes were implanted stereotactically along the longitudinal axis of each MTL [19]. The placement of electrode contacts within the hippocampus and the anterior parahippocampal gyrus, which is covered by the rhinal cortex, was ascertained by magnetic resonance imaging in each patient. For each patient, one contact within the rhinal cortex, one within the anterior part (anterior third), and one within the posterior part of the hippocampus (posterior third) were selected. Only invasive EEG recordings of the MTL contralateral to the zone of seizure origin were analyzed. These data were compared with the central electrode of scalp EEG (C3/C4) ipsilateral to the nonepileptic MTL.

Visual sleep stage scoring was carried out for each 20-second epoch according to Rechtschaffen/Kales criteria [20] by two experts. Subsequently, epochs were divided into the following categories: waking state, REM sleep, and non-REM sleep. All EEG epochs were visually inspected for movement artifacts and epileptiform activity. Epochs containing artifacts were discarded irrespective of the duration of artifacts. Furthermore, all epochs with power values above 50 *μ*V^2^ in the upper *γ* band (36–44 Hz) were discarded, to avoid high-frequency contamination, which may survive visual artifact rejection. In total, 53.0% of all EEG epochs were excluded from further analysis (45.1% based on step one, 7.9% based on step two).

Power spectra of all artifact-free epochs were calculated for each 20 seconds epoch. To increase statistical reliability of power estimates, we partitioned each 20 seconds EEG epoch into 16 nonoverlapping subsegments of 1.25 seconds duration. We used a fast Fourier transform (cosine windowing), and the frequency range was divided up into the following bands: *δ* (1–4 Hz), *θ* (4–8 Hz), *α* (8–12 Hz), *β*_1_ (12–16 Hz), *β*_2_ (16–20 Hz), *γ*_1_ (20–28 Hz), *γ*_2_ (28–36 Hz), and *γ*_3_ (36–44 Hz). Pearson's correlations between power values for scalp EEG (C3/C4) and all three locations of the medial temporal depth electrodes were calculated. Correlation values were Fisher *z*-transformed, and group differences against zero were evaluated with two-tailed *t*-tests.

## 3. RESULTS

**Figure 1:**
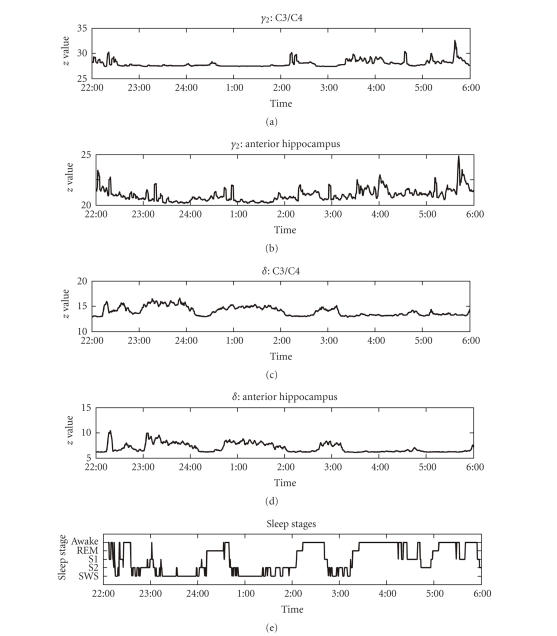
Time course of *γ*_2_ and *δ* band activity in scalp EEG and in the anterior hippocampus and sleep stages during one night in one exemplary subject. S1: sleep stage 1, S2: sleep stage 2, SWS: slow wave sleep.

**Figure 2:**
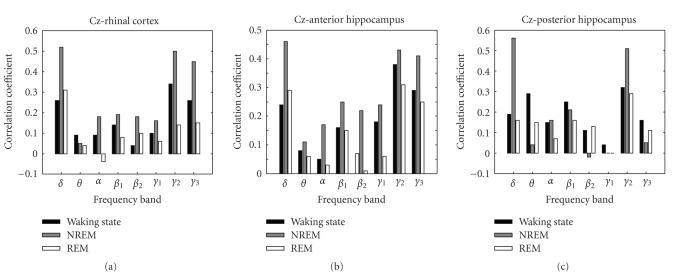
Pearson's correlation coefficient (Fisher *z*-transformed) between power densities in scalp EEG (C3/C4) versus medial temporal locations; averages across subjects are depicted. Both in the *δ* and in higher (*α* and *γ*_2_) frequency range, correlation values were maximal during NREM sleep.

Figure 1 presents raw data from one exemplary subject. Visually, there appeared to be an increased correlation of *δ* band activity during non-REM sleep. To quantify the effect of different sleep stages on interactions of EEG dynamics within neocortex and MTL, we performed a three-way ANOVA with “locus” (C3/4 compared to rhinal cortex, anterior hippocampus, or posterior hippocampus) and “sleep stage” (waking state, REM-sleep, and non-REM sleep) as repeated measures and “frequency band” as independent variable. Figure 2 depicts average values of Fisher *z*-transformed correlation values for the different frequency bands during waking state, REM-sleep, and non-REM sleep. We observed a highly significant effect of “sleep stage” (F_2,144_ = 6.49; *P* = 0.002) and a near significant effect of “frequency band” (F_7,72_ = 2.13; *P* = 0.051), but no effect of “locus” and no interaction. This result indicates that interactions of EEG dynamics within neocortex and MTL depended significantly on sleep stage. Neocortical and medial temporal EEG oscillations were more closely correlated during sleep than during waking state.

Although we did not find a significant “sleep stage” × “frequency band” interaction, we were interested in frequency-specific effects and thus conducted two-way ANOVAs with “locus” and “sleep stage” (waking state, REM sleep, and NREM sleep) as repeated measures separately for the different frequency bands. We found a significant effect of “sleep stage” (F_2,18_ = 6.00; *P* = 0.010) and a trend for a “sleep stage” × “locus” interaction (F_4,36_ = 2.14; *P* = 0.096) in the *δ* range, but not in any other frequency bands. To identify differences between pairs of sleep stages in the different frequency bands, we conducted two-way ANOVAs with “locus” and “sleep stage” (either waking state and REM sleep; or waking state and NREM sleep; or REM sleep and NREM sleep) as repeated measures separately for the different frequency bands. In the *δ* band, we found a significant effect of “sleep stage” for the comparison of waking state and NREM sleep (F_1,9_ = 7.11; *P* = 0.026). While there was no significant difference between waking state and REM sleep, the comparison of REM sleep and NREM sleep also revealed a significant effect of “sleep stage” (F_1,9_ = 7.95; *P* = 0.020) and a significant “sleep stage” × “locus” interaction (F_2,18_ = 3.99; *P* = 0.037). We thus calculated separate one-way ANOVAs for the different pairs of electrodes. We found a significant difference between NREM and REM sleep at the posterior hippocampus-Cz pair (F_1,9_ = 16.76; *P* = 0.0027), and trends for the anterior hippocampus-Cz pair (F_1,9_ = 4.27; *P* = 0.0687) and the rhinal-Cz pair (F_1,9_ = 3.56; *P* = 0.091).

Besides these effects in the *δ* frequency range, we also conducted separate two-way ANOVAs with “locus” and pairs of “sleep stage” as repeated measure in the other frequency bands. We found a trend for a difference between NREM and REM sleep both in the *α* (F_1,9_ = 3.83; *P* = 0.0819) and in the *γ*_2_ range (F_1,9_ = 3.39; *P* = 0.0987).

**Figure 3:**
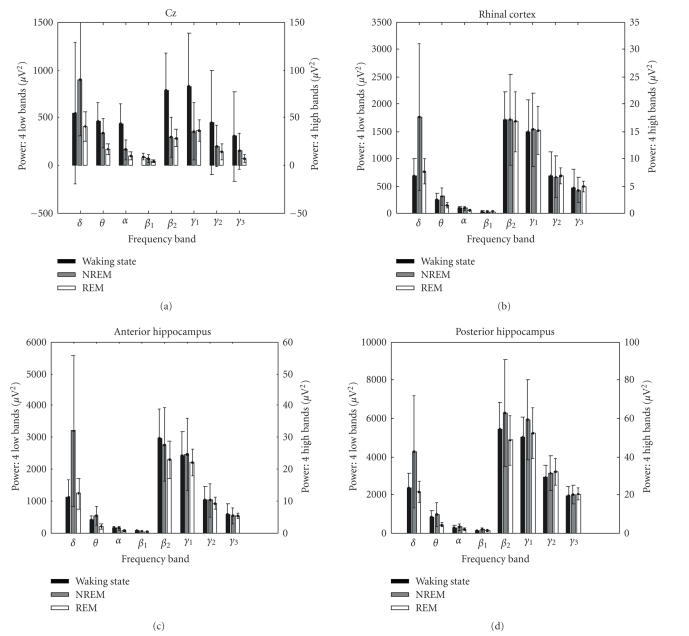
Power values (mean and standard deviation across epochs) in scalp EEG and medial temporal locations. Please note that to improve visibility of effect of sleep stage, ordinate scaling differs for low- (*δ* to *β*_1_) and high- (*β*_2_ to *γ*_3_) frequency bands. In contrast to the effect of sleep on power correlations (see Figure 2), power values in the high-frequency band were maximal during waking state.

It might be argued that the effect of sleep stage on power correlations is contaminated by differences of power in the different sleep stages. Power values depend on sleep stage, and thus in stages with low EEG power in a given frequency band, the noise may be too large to detect the correlation. In other words, the increased correlation in the *δ* band during NREM sleep as compared to waking state might be related to the fact that *δ* power is maximal during NREM sleep. However, it is unlikely that the effect of sleep stage on correlation observed in our data depends on power values for the following reasons. In the three-way ANOVA with “sleep stage” and “locus” as repeated measures and “frequency band” as independent variable reported above, we observed a main effect of “sleep stage” but no “frequency band” × “sleep stage” interaction, indicating that the effect of sleep stage did not depend on frequency band. Indeed, we did not only observe increased correlations during NREM sleep as compared to waking state in the *δ* range, but also trends for increased correlations in the *α* and *γ*_2_ range, which have a lower power during NREM sleep than waking state. The effects of sleep stage on power values are usually substantially different. *δ* and *θ* power increase during NREM sleep as compared to waking state, whereas power in higher frequency ranges decreases. This typical relationship occurred in our data as well (see Figure 3). A three-way ANOVA of power values with “sleep stage” and “locus” as repeated measures and “frequency band” as independent variable revealed a significant “sleep stage” × “frequency band” interaction (F_14,144_ = 9.771; *P* < 10^−10^; *ε* = 0.707), indicating that sleep had different effects on power in the different frequency bands.

To directly assess whether the effect of “sleep stage” on the correlation of power values depends on differences in power, we calculated the correlation of (Fisher *z*-transformed) correlation values with power across regions and frequency bands. None of these correlations reached significance (Pearson's correlation values were <0.2 in each test, corresponding to *P* values >0.6).

## 4. DISCUSSION

Our findings indicate that oscillations within the MTL and the neocortex are more closely correlated during sleep, in particular during non-REM sleep, than during waking state. This is consistent with the hypothesis that encoding of novel inputs into long-term memory, which occurs mainly during waking state, requires that medial temporal EEG dynamics are rather independent from neocortical dynamics [3], with the exception of interactions in the *θ* and *γ* range (e.g., [12, 14–16]. On the other hand, the consolidation of declarative memories during sleep may demand closer correlation of neocortical and medial temporal EEG dynamics [3], not only in the *γ* range, but also with respect to *δ* and *β* (spindle) oscillations (e.g., [17, 18]). Interestingly, correlations in the spindle frequency range only reached significance during slow-wave sleep, but not during the entire period of non-REM sleep. This might suggest that neocortical-medial temporal interactions in this frequency range are less prominent in sleep stage 2, which is most commonly associated with sleep spindles, than in deeper sleep stages.

Even though we only analyzed data from the hemisphere contralateral to the seizure onset zone, a relatively large number of epochs (53%) contained at least one epileptiform event or a movement artifact. All EEG epochs were inspected twice for movement artifacts and epileptiform activity. Artifact segments were discarded irrespective of artifact duration; for example, if a single spike or movement artifact occurred during a 20-second epoch, the entire epoch was discarded because artifacts might otherwise spuriously contribute to power estimates. As a result, the number of discarded epochs was relatively large; however, because we analyzed EEG during entire nights, the remaining data set was still extensive (mean ± std.: 178.3 ± 117.3 minutes per night).

Activity in the frequency range between 0.5 Hz and 1 Hz, that is, below the *δ* frequency range, has been termed “slow activity” (SA) and is probably due to different mechanisms than *δ* activity [21]. In principle, it would have been interesting to analyze correlation of SA between the neocortex and the MTL as well. However, the subsegments of 1.25 seconds durations which were used to analyze power values (see Methods) contain only a single cycle, or even less, of 0.5–1 Hz activity, which would have resulted in imprecise estimations of power values. We thus decided to omit this frequency range.

Previous studies on correlation of activity between hippocampus and neocortex found that hippocampal *θ* oscillations occurred in brief bursts and were most abundant during REM sleep, where they were independent of neocortical *θ* band activity [22]. While they were virtually absent during SWS, there were also longer *θ* bursts during transition from REM sleep to waking state, which occurred simultaneously with neocortical *θ* activity. These results are somewhat different from our findings that coupling was most pronounced during NREM sleep. However, it should be noted that different measures were used. While we calculated correlations of power values across 20 seconds epochs, Cantero and colleagues [22] analyzed partial directed coherence. In another study, Cantero et al. [23] reported a decrease of cortico-hippocampal coherence during sleep in a *γ* band (35–58 Hz) corresponding roughly to (but exceeding) our *γ*_3_ band (36–44 Hz). In our study, the average correlation in the *γ*_3_ range between Cz and the posterior hippocampus was also higher during waking state than during NREM sleep (see Figure 2), although this difference did not reach significance and was opposite between Cz and the other MTL locations. Further research is necessary to explain these divergent findings.

In particular, it would be interesting to investigate cortico-hippocampal coupling during replay of previously acquired information similar to results from animal studies. In rats, place cells in the hippocampus represent spatial positions by their firing rate [24]. Various studies found that during sleep periods following exploration of new environments (and thus following development of new place representations; [25]), these activity sequences are being replayed [26, 27]. Most importantly, such replay has been observed not only in the hippocampus, but also simultaneously in the neocortex as well [28]. In humans, stimulus-specific activity has only been observed in the hippocampus [29], but not in the neocortex. It is unknown, however, if this activity is replayed during consecutive sleep periods. Such relative preservation of the mechanisms underlying memory consolidation across species might be suggested by studies showing that medial temporal high-frequency bursts (“ripples”), which appear to correspond to condensed information replay [12], are coupled to neocortical sleep spindles both in rats [30–32] and humans [33].

In our analyses, we assessed functional connectivity by measuring the correlation of power values averaged across episodes of 20 seconds. While this approach lacks temporal resolution, it allows to clearly assign correlation values to specific sleep stages. Of course, correlations may depend on the investigated timescale. In principle, an evaluation of correlations at shorter time scales might allow for more mechanistic interpretations. Here, we intended to analyze state-related correlations across 20 seconds epochs defined by Rechtschaffen and Kales criteria [20], because classification of sleep stages on a smaller time scale has not been validated. Previous bivariate measures of intracranial EEG data utilized either power correlation [34, 35] or phase synchronization [14, 36]. The latter approach is particularly well suited to study interactions of intracranial EEG with a high temporal resolution. However, phase synchronization between scalp and intracranial EEG is problematic because of the different properties of scalp and intracranial EEG, and because scalp activity is transferred through structures with strong low-pass filtering properties such as bone and skin [37], which may lead to phase distortions. In contrast, the reported analysis of power correlation is probably less hampered by these difficulties. Further recordings in patients with both medial temporal depth electrodes and subdural electrodes are required to calculate phase synchronization during different sleep stages.

Taken together, our findings support the idea that medial temporal and neocortical dynamics are more integrated during sleep, in particular NREM sleep, than during waking state [38].
